# Parafoveal Processing of Orthography, Phonology, and Semantics during Chinese Reading: Effects of Foveal Load

**DOI:** 10.3390/brainsci14050512

**Published:** 2024-05-18

**Authors:** Lei Zhang, Liangyue Kang, Wanying Chen, Fang Xie, Kayleigh L. Warrington

**Affiliations:** 1 Intelligent Laboratory of Child and Adolescent Mental Health and Crisis Intervention of Zhejiang Province, Zhejiang Normal University, Wucheng Distract, Jinhua 321004, China; psyzhanglei@zjnu.edu.cn (L.Z.); psykangly@zjnu.edu.cn (L.K.); cwypsy21@zjnu.edu.cn (W.C.); 2 School of Psychology, Zhejiang Normal University, Wucheng District, Jinhua 321004, China; 3 School of Psychology and Vision Sciences, University of Leicester, George Davies Centre, Lancaster Road, Leicester LE1 7HA, UK

**Keywords:** foveal load hypothesis, parafoveal preview, Chinese reading, eye movements, word recognition

## Abstract

The foveal load hypothesis assumes that the ease (or difficulty) of processing the currently fixated word in a sentence can influence processing of the upcoming word(s), such that parafoveal preview is reduced when foveal load is high. Recent investigations using pseudo-character previews reported an absence of foveal load effects in Chinese reading. Substantial Chinese studies to date provide some evidence to show that parafoveal words may be processed orthographically, phonologically, or semantically. However, it has not yet been established whether parafoveal processing is equivalent in terms of the type of parafoveal information extracted (orthographic, phonological, semantic) under different foveal load conditions. Accordingly, the present study investigated this issue with two experiments. Participants’ eye movements were recorded as they read sentences in which foveal load was manipulated by placing a low- or high-frequency word N preceding a critical word. The preview validity of the upcoming word N + 1 was manipulated in Experiment 1, and word N + 2 in Experiment 2. The parafoveal preview was either identical to word N + 1(or word N + 2); orthographically related; phonologically related; semantically related; or an unrelated pseudo-character. The results showed robust main effects of frequency and preview type on both N + 1 and N + 2. Crucially, however, interactions between foveal load and preview type were absent, indicating that foveal load does not modulate the types of parafoveal information processed during Chinese reading.

## 1. Introduction

Extensive research using the gaze-contingent boundary paradigm [1] has established the crucial role of parafoveal processing in normal reading. By manipulating the availability of valid parafoveal information, researchers have uncovered that fixation durations on critical words are shorter following valid parafoveal previews, compared with invalid preview conditions in which parafoveal information is masked. This effect, termed the parafoveal preview benefit [2], demonstrates that information extracted parafoveally facilitates processing on the subsequent fixation [3], and therefore aids efficient processing. Research has also aimed to understand the factors that influence the ability to effectively process parafoveal information. One hypothesis that the ease (or difficulty) with which the currently fixated word is processed may influence the processing of parafoveal word(s) known as the foveal load effect. This effect was initially demonstrated by Henderson et al. [4], who found that when the fixated word is more difficult to process, such as when it is low frequency, fewer attentional resources are available to process upcoming words, therefore hindering parafoveal processing.

However, subsequent research has produced a mixed picture regarding the existence of a foveal load effect. A meta-analysis [5] found only six out of sixteen published studies demonstrated foveal load effects. There are three important considerations regarding the interpretation of existing research, which need to be resolved in order to truly understand the nature of foveal load effects in reading. These are: (1) the role of writing script, (2) establishing appropriate neutral parafoveal mask, and (3) establishing how foveal load may influence parafoveal processing, in terms of both the amount and type of information extracted from the parafovea. It is these three issues that we aim to tackle in the current study.

First, considering the role of writing script, current knowledge of foveal load effects is based almost exclusively on alphabet languages. Indeed, all studies included in Veldre et al.’s [5] meta-analysis explored only Latinate alphabetic reading (with the exception of one study that manipulated N + 1 load). However, there is good reason to speculate that script-specific processing demands may shape foveal load effects. Here, we focus on Chinese. Chinese is visually dense, with most words comprising one or two-characters [6]. As a result, upcoming words fall closer to fixation [7], allowing for more extensive extraction of parafoveal information [8,9]. Moreover, as written Chinese is unspaced, readers must segment words from the character’s string; therefore, they may prioritize the allocation of their attention to upcoming characters more than readers of alphabetic languages [10]. Indeed, parafoveal preview appears to be more substantial, and processing of word N + 2 appears to be more common in Chinese, compared with alphabetic reading [11]. These script-based differences may modulate the influence of foveal load, such that parafoveal processing in Chinese reading may be more robust and less susceptible to foveal load effects. Indeed, while there have been very few investigations of foveal load effects in Chinese reading, evidence to date suggests that foveal load effects may be absent. Zhang et al. [12] examined effects of frequency-based foveal load on the processing of word N + 1. They found robust N + 1 processing in all conditions, and no evidence of a foveal load effect. As more efficient parafoveal processing during Chinese reading may allow the one-character N + 1 to be pre-processed relatively easily even when foveal load is high. Lv et al. [10] investigated whether foveal load influenced the preview benefit of word N + 2. The results also revealed robust N + 2 processing in both conditions and no interactive effects. These findings provide evidence that foveal load does not affect Chinese reading, and upcoming words can be parafoveally processed effectively under both high and low foveal load.

To ensure that any differences observed across conditions are the result of differences in parafoveal processing, it is important that the parafoveal mask is neutral. In their meta-analysis, Veldre et al. [5] hypothesized that the visually/orthographically unusual baseline preview conditions used in many studies may interfere with normal processing and contribute to mixed results found in alphabetic studies. In two experiments, they found a foveal load effect only when the preview consisted of a random consonant string, but found no interactions when using an alternating case preview or a nonword neighbor preview. They argued that interactive effects were driven by preview cost from the perceptually distinct illegal nonword preview. Further evidence that interactive effects reflect the presence of perceptually distinct parafoveal previews comes from Findelsberger et al. [13], who found a foveal load effect with visually degraded previews, but not traditional letter previews. Visual degradation increased display change awareness and may capture the reader’s attention, leading to additional costs similar to illegal nonword previews [14].

In the current study, we adopt unrelated pseudo-character previews as our baseline. Pseudo-characters are formed from components found in Chinese characters, but are not real characters. As such, they are designed to provide no meaningful information, while looking natural and not perceptually distinct from real characters. Studies using pseudo-character previews typically report low levels of display change awareness, suggesting that they do not capture attention in the same way as illegal letter strings or degraded previews [10,15]. Unrelated character previews that use real, legal characters may offer the opportunity for readers to activate and begin processing this irrelevant information and any processing of this unrelated character will then subsequently need to be corrected [16]. We therefore consider the pseudo-character to be a good candidate for offering a truly neutral (not strongly associated with either disruption or facilitation) baseline. Pseudo-characters are also the mask type adopted by previous Chinese studies of foveal load, where the effect has been absent.

This adoption of an effective neutral baseline mask offers an important opportunity to further explore and consider the role of preview type in understanding foveal load effects.

Crucially, manipulations of foveal load have the potential to modulate parafoveal processing in two ways: by affecting the spatial extent of processing, or the linguistic depth of processing. Depth of processing can be considered a measure of the degree to which the parafoveal word has been efficiently processed. The majority of studies to date provide an estimate of processing depth by comparing the size of the preview benefit for an identical preview relative to a (typically orthographic) mask to infer the depth of processing, such that it is assumed that if the preview benefit across conditions is similar, a similar amount of processing must have been undertaken. One way of characterizing linguistic depth is in terms of the types of information that can be extracted parafoveally, that is, the degree to which a parafoveal word may be processed orthographically, phonologically, or semantically. Therefore, a similar preview benefit in these circumstances does not necessary mean that the processing undertaken across conditions is equivalent, as such an approach does not provide a measure of what type of information has been processed. Importantly, then, while pseudo-characters seem to offer an effective neutral baseline, because pseudo-characters do not contain meaningful information, studies that use only a pseudo-character condition cannot establish whether parafoveal processing is equivalent in terms of the type of information processed across foveal load conditions. Here, our aim is to explore more directly the types of linguistic information that can be parafoveally extracted under high and low foveal load conditions, as this will offer more nuanced understanding.

The “orthography-to-phonology-to-semantics” route in word recognition [17] predicts that orthographic information is accessed earlier during the time course of processing than phonological information, which in turn is accessed prior to semantic information. Following these assumptions, evidence of the parafoveal preview benefits for phonology or semantics indicates greater depth of parafoveal processing, as more information has been extracted. In line with this, in English, there is robust evidence that orthographically related [18,19,20,21] and phonologically related [22,23] words in place of N + 1 yield strong preview benefits, indicating that readers extract orthographic and phonological information parafoveally. Parafoveal processing of high-level information such as semantics, on the other hand, appears to be uncommon in English [3,24,25,26], and where such effects are observed [27,28,29], they may be determined by the plausibility of the preview given the sentence context, rather than the relationship between the preview and the target (i.e., plausibility preview effects [30,31,32]). This suggests that English words are typically not pre-processed with sufficient depth to access semantic information, while words in German, a language with a shallow orthography, may allow for more frequent parafoveal semantic processing (Hohenstein et al. [33], but see Rayner et al. [34] for evidence that this may be driven by German noun capitalization).

In contrast to Latinate alphabetic languages, Chinese characters are mapped more closely to meaning than to phonology [35]. As a result, skilled Chinese readers may have more direct access to semantics from orthography, and so the “orthography-to-phonology-to-semantics” route may be less dominant, and readers’ information processing priority may emphasize semantics over phonology [36]. In support of this, robust parafoveal extraction of semantic information has been observed in Chinese, while phonological preview benefit appears to be a small and occurs at a relatively late stage of activation [16,23,37]. The demands of the language may therefore shape the information processing priority, and so what constitutes efficient, or deep, parafoveal processing depends on the language being studied. Moreover, the visual characteristics of written Chinese, which may promote more extensive parafoveal processing [11] make it the ideal language to observe subtle differences in processing type as a result of foveal load.

M. Yan et al. [38] reported that in Chinese, semantic preview benefit is larger when pre-boundary fixations are shorter (perhaps indicating greater ease of processing), while phonological benefit is larger when pre-boundary fixations are longer (perhaps indicating greater processing difficulty). Developing readers show early activation of phonology, which diminishes with the development of reading skills [39], while L2 learner’s parafoveal processing appears limited to low-level orthographic features [40]. Taken together, these studies indicate that the types of information processed parafoveally can differ across conditions/readers, such that linguistic depth of processing may not be equivalent, even when substantial preview benefit is observed in both conditions. There is some evidence that the presence of a semantic preview benefit may be a marker of efficient parafoveal processing, and so exploring the type of information extracted from the parafovea under different foveal load conditions might be informative about the efficiency of processing. This study therefore provides the first comprehensive assessment of whether foveal load modulates preview benefit from orthographically, phonologically, and semantically related masks. We adopt an approach of comparing the preview benefit for related preview masks compared to the unrelated baseline. Similar approaches have been used in numerous studies to establish what types of information can be parafoveally extracted [16,36]. Moreover, parafoveal processing, as a fundamental process, may be more robust in Chinese reading, and it is reasonable to speculate that any influence of foveal load may be more likely to influence the type of information processed (rather than the presence of parafoveal processing).

This issue has important implications for models of eye movement control during Chinese reading. The Chinese Reading Model (CRM [41]) predicts foveal load effects on parafoveal processing, but it only directly predicts them on the basis of evidence that saccades are longer when leaving high-frequency words than low-frequency words. Note, that several studies demonstrate that foveal load influences saccade targeting [42], but this influence appears to occur independent of parafoveal preview [12] and does not reflect reduced parafoveal processing. E-Z reader [43] and SWIFT (saccade-generation with inhibition by foveal targets) [44] also incorporate foveal load effects into their basic architecture, with the E-Z reader predicting effects on linguistic depth, while SWIFT predicts an effect on spatial extent, although neither make a specific prediction regarding foveal load in Chinese reading. Therefore, further research is needed to clarify the nature of foveal load effects and inform the future development of Chinese reading models. Understanding the influence of foveal load on parafoveal processing can optimize reading strategies and enhance reading comprehension and literacy instruction.

Above all, previous studies on the impact of foveal load on parafoveal processing depth have not considered the potential differences in the type of information processed under the conditions of high or low foveal load. Thus, the current study examines whether foveal load may modulate linguistic depth of parafoveal processing during Chinese reading, such that the types of parafoveal information extracted may differ as a function of foveal load. Experiment 1 examines the processing of word N + 1. Given the robust N + 2 preview benefits observed in Chinese reading, Experiment 2 additionally explores the processing of N + 2. Accordingly, sentences were created in which three consecutive words were manipulated, we refer to these as word N (the foveal word), word N + 1 (one-character word), and word N + 2 (one-character word). Word foveal load was manipulated using word frequency. The preview validity of word N + 1 (Experiment 1) or word N + 2 (Experiment 2) was manipulated with five preview types: identical, orthographically related word, phonologically related word, semantically related word, and unrelated pseudo-character.

Following previous research, we anticipated the main effects of foveal load on word N and effects of preview validity on word N + 1/N + 2. Given the substantial parafoveal processing observed in Chinese, we expect that readers will benefit from the availability of an orthographically, phonologically, or semantically related preview of N + 1, compared with the unrelated pseudo-character. As little research has investigated the depth of processing for N + 2, the predictions here are more tentative, but we anticipate robust N + 2 processing (at least in the low-load condition). Crucially, if there are interactive effects of foveal load and preview type, this would provide novel evidence that foveal load modulates parafoveal processing in Chinese reading. Given the findings of Zhang et al. [12] and Lv et al. [10], we predict additive effects of preview and frequency for comparisons of an identical preview with a pseudo-character preview. On this basis, we also anticipate an orthographic preview benefit in both conditions. If fewer attentional resources are allocated to the parafovea when foveal load is high, readers may be less likely to benefit from semantic information in this condition.

In order to investigate whether foveal processing load affected the depth of preview benefit for the subsequent word N + 1, we used a boundary paradigm and manipulated foveal load and different parafoveal preview types at the word N + 1 position. Experiment 1 adopted a 2 (foveal load: high-frequency, low-frequency) × 5 (preview type: identical, orthographically related, phonologically related, semantically related, and pseudo-character) within-subject design. Given that Chinese readers could access the information of parafoveal word N + 2 (when N + 1 is a single word), Experiment 2 further examines whether preview benefit for word N + 2 was reduced under conditions of increased foveal processing difficulty. Experiment 2 used the same experimental design as Experiment 1.

## 2. Experiment 1

### 2.1. Method

This research was approved by the research ethics committee of the Zhejiang Normal University (IRB approval number: ZSRT2023144) and conducted in accordance with the principles of the Declaration of Helsinki.

#### 2.1.1. Participants

Participants were 150 adults (i.e., over age 18) aged 18–24 years (M = 20.3 years) from the authors’ University. Six participants who reported seeing more than 10% display changes were excluded, leaving 144 participants for analysis. All participants were native Chinese speakers with normal or corrected-to-normal vision (i.e., greater than 20/40 vision in Snellen values) using a Tumbling E eye chart [45], and read several hours per-week. We calculated experimental power for an effect size of d = 0.43 [10] using software from Westfall et al. [46]. Our analysis indicated that at least 120 participants are required for 60 stimuli to achieve 0.80 power [47]. Therefore, our sample size of 144 participants exceeds the minimum required. Informed consent was obtained from all participants.

#### 2.1.2. Apparatus and Procedure

Eye movements were recorded using an Eyelink portable duo at a sampling rate of 2000 HZ. Viewing was binocular, but only the right eye was tracked. Sentences were displayed as black text on a grey background in Song font on a 24-inch-high monitor (1920 × 1080 resolution, 144 HZ refresh rate). At a 60 cm viewing distance, each character subtended approximately 1°, and so was of normal size for reading.

Participants took part individually, and were instructed to read normally and for comprehension. Prior to the presentation of the first sentence, a three-point horizontal calibration and validation of calibration accuracy procedure was checked (maintaining spatial accuracy of 0.30° or better). At the start of each trail, a fixation cross equal in size to one character space was presented on the left side of the screen. Once the participant fixated this cross, the sentence was presented with the first character replacing the cross. Participants pressed a response key to terminate the display once they finished reading. A total of 30% experimental sentences followed by a comprehension question requiring a yes/no response. At the end of the experiment, participants were asked if they noticed any display changes. The participants who reported seeing more than 10% display changes were excluded. Each participant completed ten practice sentence and then read sixty experimental sentence and forty filler sentences at random. The whole experiment lasted approximately 30 min.

#### 2.1.3. Stimuli and Design

**Figure 1:**
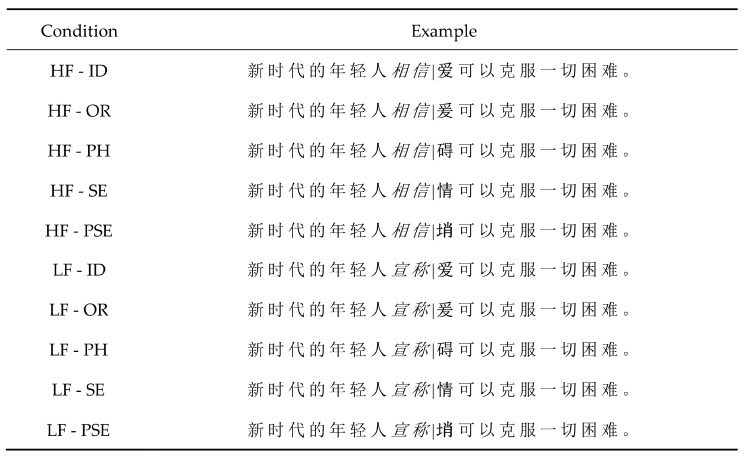
An example of sentence materials in Experiment 1. Note. HF = high frequency; LF = low frequency; ID = identical preview; OR = orthographically related preview PH = phonologically related preview, SE = semantically related preview, PSE = pseudo-character preview, unrelated to target word. The pretarget word (N) is presented in italics, while the previews of the target (N + 1) is in bold (for illustration purposes only). The vertical black line represents the position of the invisible boundary. As readers’ eyes crossed the boundary, the preview was replaced by the target. High and low refer to the foveal load condition. The sentence for HF condition means ‘Young people in the new era believe that love can overcome all difficulties’. The sentence for LF condition means ‘Young people in the new era claim that love can overcome all difficulties’.

Stimuli consisted of 60 sentence frames in which we embedded one pretarget word (referred to word N) and one target word (referred to as word N + 1). Sentences were 16–25 characters in length (M = 19.60, SD = 2.35). The pretarget and target words never appeared among the first three or the last three words in the sentence. Foveal load was manipulated by making the pretarget word either high or low frequency. In total, 120 two-character words were selected from Cai et al. [48], of which 60 were high-frequency (M = 411.57 words per-million, SD = 103.99) and 60 were low-frequency (M = 22.18 words per-million, SD = 5.42), F (1,59) = 14.66, *p* < 0.001. Low- and high-frequency words were closely matched in visual complexity, F (1, 59) = 1.15, *p* = 0.29. The boundary paradigm was used to manipulate the parafoveal preview of word N + 1 in five preview conditions (see Figure 1): (1) identical to the target word, (2) orthographically related to the target word, (3) phonologically related to the target word, (4) semantically related to the target word, and (5) pseudo-character unrelated to the target word. The orthographically related, phonologically related, and semantically related words were matched with the target word in terms of character frequency (F (3, 57) = 1.94, *p* = 0.14) and number of strokes (F (3, 57) = 0.54, *p* = 0.66).

A group of 15 undergraduate students were asked to rate the orthographic, phonological, and semantic relatedness between the target and each of the four preview characters on a seven-point scale (1 = highly unrelated; 7 = highly related) to ensure that the preview characters were related to the targets only on the desired dimensions (see Table 1). The pseudo-characters were created with the Character Editor on a Windows 10 system laptop. They did not share any semantically, phonetically, or visually similar radicals with the corresponding target word, but were matched to the target word in relation to its stroke number (F (1, 59) = 0.44, *p* = 0.51).

A cloze task with a non-overlapping group of 21 participants who did not take part in the eye tracking study evaluated the naturalness and plausibility of the sentences on a seven-point scale. Rating did not differ between low- and high-load conditions for naturalness (M = 6.06, F (1,59) = 2.93, *p* = 0.09) or plausibility (M = 5.95, F (1,59) = 0.11, *p* = 0.74). To assess the predictability of the pretarget words, a group of 10 different participants provided cloze norming data for word N. They were given each sentence frame up to and including word N-1 and asked to provide the word that was most likely to come next. Additionally, the mean predictability and plausibility of the preview words were also assessed with another 20 different participants (10 for predictability, 10 for plausibility), and there were not any significant differences between the low- and high-load conditions (Fs (1,59) < 2.10, Ps > 0.05). A further 20 participants rated the predictability of the target word N + 1 by performing the cloze task for the sentence content up to N + 1. The mean predictabilities of the pretarget word (N) and the target word (N + 1) were very low, without any significant differences between low- and high-load conditions (for pretarget words, F (1,59) = 2.08, *p* = 0.16; for target words, F (1,59) = 1.89, *p* = 0.17).

Ten lists were constructed. Each list included sixty experimental sentences with six sentences from each condition. A Latin-square design ensured that participants saw each target word only once, with an equal number of items in each condition.

#### 2.1.4. Data Analysis

Comprehension accuracy was high (M = 91.8%) for all participants, indicating that participants understood the sentence well. Following standard procedures, fixations shorter than 80 ms or longer than 1200 ms were removed (affecting 2.5% of fixations). We excluded trials in which a saccade triggered a display change but terminated to the left of the target word, or display change triggered by fixations before the boundary were excluded (i.e., a ‘j-hook’, 7.9% of trials) and display change that was completed more than 10 ms after the onset of subsequent fixation (following Slattery et al. [49], 2.3% of trials). The boundary cleaning procedure was conducted using an R script adapted from Drieghe et al. [50].

The remaining data were analyzed with linear mixed-effects models (LMMs or generalized (G)LMM for binomial variables; Baayen et al. [51]), conducted using lme4 package [52] in R (version, 4.3.1; R Core Team [53]). A maximal random effects structure was used where possible with participants and sentences as crossed-random effects [54]. If the maximal model did not converge, we used principal components analysis (PCA) and dropped random components that generated the smallest variances until the model converged. Analyses for both untransformed and log-transformed data produced the same patterns of results, so only results for untransformed data are reported. Pairwise comparisons of main effects and interactions were defined using the MASS package [55], and a contrast matrix was used to assess the effects of previewing. These compared pseudo-character unrelated conditions against identical conditions, pseudo-character unrelated conditions against orthographically related conditions, pseudo-character unrelated conditions against phonologically related conditions, and pseudo-character unrelated conditions against semantically related conditions. Following convention, t > 1.96 were considered significant.

Analyses for pretarget N and target N + 1 regions are reported separately. The following measures are reported: first-fixation duration (FFD, the duration of the first fixation on a word during first-pass reading), gaze duration (GD, the sum of all fixations on a word during first-pass reading), regression path duration (RPD, the sum of all fixations made before making a saccade to the right of the word, including regressions to earlier sections of text), total time (TT, the sum of all fixations on the word), and skipping probability (SKIP, probability of not fixating a word during first-pass reading).

### 2.2. Results

Means and standard deviations are shown in Table 2, and statistical analysis are summarized in Table 3.

Word N. A significant frequency effect was found in all measures such that readers produced shorter fixation times and higher skipping probabilities for high- rather than low-frequency words. These effects complement the findings from previous Chinese studies [56,57] and demonstrate that the manipulation of foveal load was effective. However, there were no effects of word N + 1 preview on first-pass reading times on word N. More importantly, there were no interactions between foveal load and parafoveal preview. Thus, fixation durations on word N were affected by its frequency, but not by parafoveal preview type, indicating that the parafoveal-on-foveal effect was absent, in line with previous research [10,12].

**Figure 2:**
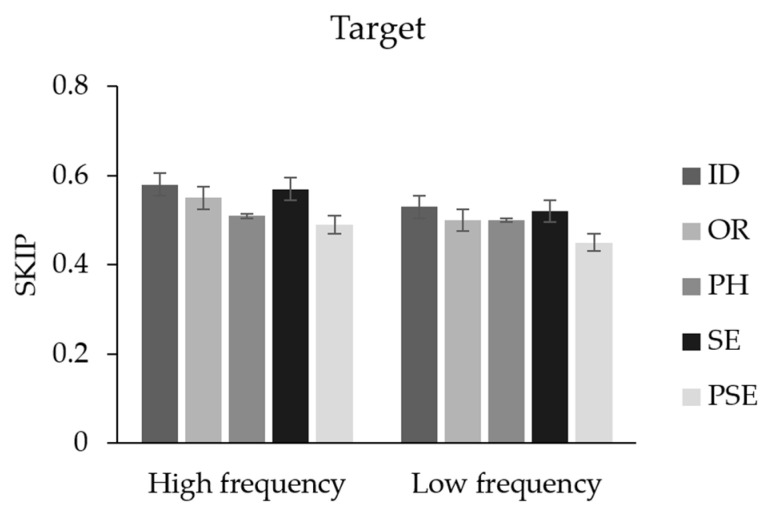
Figure shows the differences between preview types in SKIP. Note. Skipping is the best measure to show the differences between preview types.

Word N + 1. Spillover effects of word N frequency on word N + 1 were present for all measures, except FFD, such that fixation durations on word N + 1 were longer when word N was low- rather than high-frequency (Zhang et al. [12] observed similar effects on skipping rates, but not reading times). Turning to preview effects, there was a significant benefit from an identical preview relative to an unrelated pseudo-character preview on all measures. Compared with the pseudo-character unrelated preview, readers also produced shorter fixation times (in GD and TT), shorter RPD, and higher skipping rates (see Figure 2) when the preview was orthographically related to the target word. In addition, compared to the pseudo-character unrelated preview, readers produced shorter total fixation times and higher skipping rates when the preview was phonologically related to the target. Skipping rates were higher when the preview was semantically related to the target, compared with the pseudo-character preview, although there were no semantic preview benefits on fixation durations. These findings suggest that readers can acquire orthographic, phonologic, and semantic information parafoveally. More crucially, there were no significant interactions between foveal frequency and preview types for all the measures; this indicates that foveal load did not modulate processing of any parafoveal information type.

### 2.3. Discussion

In Experiment 1, we manipulated the frequency of foveal word N and parafoveal preview type of word N + 1 to examine whether foveal load modulates the type of linguistic information extracted during the parafoveal preview. For word N, fixation durations were shorter, and skipping probabilities were greater for high- compared to low-frequency words, indicating that the manipulation of foveal load was effective. Interestingly, in contrast to Zhang et al. [12], we observed spillover effects of word N frequency on N + 1 processing times, as well as word skipping rates. The clear N + 1 preview effects showed a preview benefit in reading times for orthographically and phonologically related previews (although this effect was restricted to later measures) and a benefit in word skipping rates for semantically related previews. This supports previous research demonstrating the robust extraction of parafoveal information in Chinese reading. The absence of the interaction indicated that foveal load did not influence the types of parafoveal information extracted for word N + 1. We interpret this as suggesting that substantial linguistic processing of N + 1 occurs under both high and low foveal load conditions.

Meta-analysis findings support the notion that parafoveal processing in Chinese may be more extensive than in alphabetic languages. As word N + 1 in Experiment 1 was always a single character, readers may be able to extract parafoveal information easily, regardless of foveal load. N + 2 pre-processing appears to be more common in Chinese reading, and therefore any effects of foveal load may be shifted from N + 1 to N + 2. To test this possibility, Experiment 2 examined the effect of foveal load on processing for N + 2, using the same preview types as Experiment 1.

## 3. Experiment 2

### 3.1. Method

#### 3.1.1. Participants

A separate sample of 150 young students aged 17–24 years (M = 20.1 years) from the authors’ University were recruited. Nine participants were excluded as they reported detecting more than 10% display changes, leaving 141 participants for analysis. All participants were native speakers of Chinese and were screened for normal visual acuity (corrected acuity of >20/40 in Snellen values) using a Tumbling E Eye-chart [45]. Informed consent was obtained from all participants.

#### 3.1.2. Apparatus and Procedure

The apparatus and procedure were identical to Experiment 1.

#### 3.1.3. Stimuli and Design

**Figure 3:**
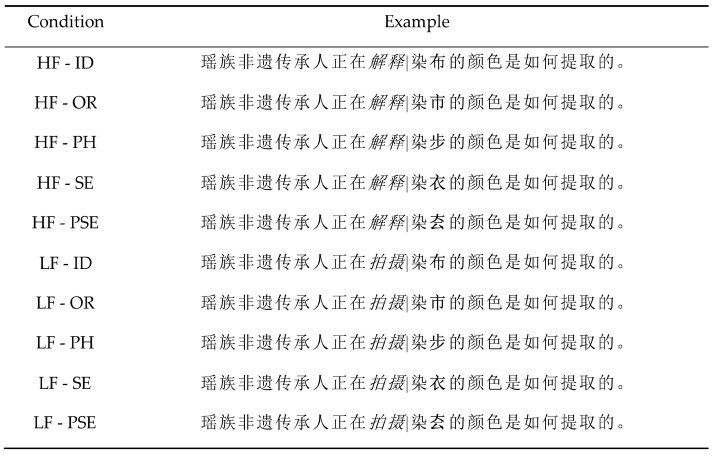
An example of sentence materials in Experiment 2. Note. HF = high frequency; LF = low frequency; ID = identical preview; OR = orthographically related preview PH = phonologically related preview, SE = semantically related preview, PSE = pseudo-character preview, unrelated to target word. The pretarget word (N) is presented in italics, while the previews of the target (N + 2) is in bold (for illustration purposes only). The vertical black line represents the position of the invisible boundary. As readers’ eyes crossed the boundary, the preview was replaced by the target. High and low refer to the foveal load condition. The sentence for HF condition means ‘The Yao ethnic heritage inheritor is explaining how the colors for dyeing cloth are extracted’. The sentence for LF condition means ‘The Yao ethnic heritage inheritor is filming how the colors for dyeing cloth are extracted’.

Following the same design as Experiment 1, the stimuli consisted of 60 sentence pairs that included a two-character pretarget word (word N) of high- (M = 320.44 words per-million, SD = 249.54) or low-frequency (M = 13.91 words per-million, SD = 15.01), F (1,59) = 90.21, *p* < 0.001. Sentences were 16–25 characters long. Both N + 1 and N+2 were single-character words. N + 1 and N + 2 cannot combine to form a two-character word. Word N + 2 was the critical word. The masking conditions were the same as in Experiment 1 (see Figure 3). Preview words were matched in terms of character frequency (F (1, 59) = 0.35, *p* = 0.79) and numbers of strokes (F (1,59) = 0.64, *p* = 0.64) with critical words. The orthographic, phonological, and semantic relatedness between the target and each of the four preview characters were rated by three different groups (15 for each) of participants on a seven-point scale (1 = highly unrelated; 7 = highly related) to ensure that the preview characters were related to the targets only on the desired dimensions (see Table 4). The unrelated preview did not share any semantically, phonetically, or visually similar radicals with the corresponding target word, but was matched to the target word in relation to its stroke number F (1,59) = 0.12, *p* = 0.73.

A cloze task with 21 participants (who did not take part in other experiments) evaluated the naturalness and plausibility of the sentences on a seven-point scale. Ratings did not differ between low- and high-load conditions for naturalness (M = 6.32, F (1,59) = 0.17, *p* = 0.68) or plausibility (M = 6.33, F (1,59) = 0.12, *p* = 0.73). To assess the predictability of the pretarget words, a group of 10 different participants provided cloze norming data for word N, using the same procedure as Experiment 1. Additionally, the mean predictability and plausibility of the preview words were also assessed with another 20 different participants (10 for predictability, 10 for plausibility), and there were no any significant differences between low- and high-load conditions (Fs (1,59) < 1.52, Ps > 0.05). A further 20 participants rated the predictability of the target word N + 2 by performing the cloze task for the sentence content up to the word N + 1. The mean predictabilities of word N and word N + 2 were very low without any significant differences between low- and high-load conditions (for pretarget words, F (1,59) = 0.20, *p* = 0.66; for target words, F (1,59) = 1.00, *p* = 0.32).

#### 3.1.4. Data Analysis

Comprehension accuracy was high (M = 94.1%) for all participants, indicating that participants read the sentences properly and understood them well. Before analyzing the data, trials were removed if they met the following conditions: they had fixations shorter than 80 ms or longer than 1200 ms (affecting 2.5% of fixations); a saccade triggered a display change but terminated to the left side of the target word, or a display change occurred before the boundary (6.8% of trials); or if the display change was completed more than 10 ms after the onset of a subsequent fixation (2.6% of trials). The same eye movement measures were analyzed as in Experiment 1.

### 3.2. Results

Means and standard deviations are shown in Table 5, and statistical analyses are summarized in Table 6.

Word N. As in Experiment 1, there were significant effects of frequency on all measures, with longer fixation times and lower skipping rates for low- compared to high-frequency words. The main effect of word N + 2 preview was significant only in total time such that readers spent longer on word N when N + 2 was the pseudo-character unrelated preview relative to the identical preview. There were no interactions between frequency and parafoveal preview types in any measure.

Word N + 2. For effects of word N frequency, RPD was longer when the pretarget word was difficult relative to when it was easy. There was no other evidence of a spillover effect of word N frequency to word N + 2, in line with previous findings [10]. Turning to the effects of previewing, fixation times (FFD, GD, TT, RPD) were longer and skipping rates were lower in the unrelated pseudo-character preview condition, relative to the identity preview condition. However, despite having slower reading times when compared with the identity condition, there was no evidence of a preview benefit from the orthographically, phonologically, or semantically related previews. In line with Experiment 1, interactions between foveal load and preview types were absent, indicating that foveal load did not modulate processing of parafoveal word N + 2.

### 3.3. Discussion

In Experiment 2, we manipulated the frequency of the foveal word N and parafoveal preview type of word N + 2 to examine whether foveal load modulates the depth of parafoveal processing. The foveal load manipulation was again effective, as fixation durations were lower, and skipping probabilities were greater for high- compared to low-frequency words. However, in comparison with Experiment 1, the effects of the preview were significant only for the comparison of identical and unrelated pseudo-character previews. There were no preview benefits for the other preview types (possible reasons for this are considered in the General Discussion). Crucially, in line with Lv et al. [10], there were no interactions between frequency and preview, demonstrating that Chinese readers undertake parafoveal pre-processing of word N + 2 in both low and high foveal load conditions.

## 4. General Discussion

In the current study, we conducted two experiments examining the nature of foveal load effects in Chinese reading. To do this, we investigated preview effects under high and low foveal processing load (manipulated using word frequency) using a range of preview types. We assessed whether foveal load modulates the linguistic depth of parafoveal processing, measured as the types of parafoveal information obtained during parafoveal preview. Experiment 1 examined these issues for Word N + 1, and Experiment 2 examined these issues for Word N + 2.

Robust preview benefit effects were observed on both N + 1 and N + 2, contributing to a growing body of evidence indicating substantial parafoveal processing in Chinese reading [11]. Moreover, Experiment 1 provided evidence to support the parafoveal extraction of orthographic, phonological, and semantic information from N + 1, in line with previous research [16,23,37,58]. The semantic preview benefit observed here was more limited than the semantic benefit reported by M. Yan et al. [16]. However, Yan et al. used only visually and structurally simple pictographic and indicative characters as targets to maximize the chances of observing a semantic preview benefit, whereas many of the characters in the current study comprised two components/radicals [59], and these more complex characters may be preprocessed parafoveally with different priority. It could be argued that because the pseudo-character does not contain any meaningful lexical content, that the difference between the unrelated and related conditions may reflect a general benefit relating to previewing a real character. However, we do not believe that this effect can fully account for the pattern of results observed, as if this were the case, we would anticipate a similar preview benefit for all related conditions relative to the unrelated conditions. This was not the case. Therefore, the differences among the related preview conditions in Experiment 1 strongly indicate that these specific linguistic characteristics were processed parafoveally. Nevertheless, there is considerable debate regarding the most suitable “neutral” baseline mask type [11], and so the potential role of mask type should be further explored in future.

The pattern for Experiment 2 was slightly less clear. While the increase in fixation times for pseudo-character previews relative to identical previews provides strong evidence of N + 2 preview effects, readers did not benefit from the orthographic, phonological, or semantic relatedness of the masking character relative to the valid character. One possible explanation is that this preview benefit effect primarily reflects a preview cost associated with the unrelated pseudo-character mask. The large preview effects seen here in comparison with previous research [10,60,61] may support such an interpretation. However, it should also be noted that reading times for all preview types (not only the unrelated pseudo-character preview) were substantially increased relative to the identical preview condition. Moreover, display change awareness was low, and although the pseudo-characters did not provide useful preview information, they were designed to be character-like and not perceptually distinct, although, as noted above, we acknowledge that the role of mask type warrants further consideration. Alternatively, it may also be the case that the benefit obtained from mask-target relatedness for word N + 2 is much more subtle and/or less consistent than for N + 1, and so was missed in the current study. Indeed, when considering the pattern of effects observed in Experiment 1, only orthographically related previews produced a robust benefit on early fixation measures, while the effects of phonologically related previews were restricted to total time and semantically related previews were restricted to word skipping. Word skipping for N + 2 may be a less sensitive measure of parafoveal extraction for two reasons. Firstly, following standard procedures, analyses of word N + 2 include both instances where N + 1 was fixated and instances where N + 1 was skipped [60], and so skipping of N + 2 is likely to also be affected by processing that occurs at N + 1. Second, as N + 1 and N + 2 formed separate words, a skipping decision programmed on word N would involve skipping two consecutive words. Therefore, the opportunity to observe these preview benefits may have been limited. Further research is therefore needed to more fully characterize the nature of N + 2 parafoveal processing in Chinese reading. However, it remains clear that substantial N + 2 pre-processing is undertaken both when foveal load is low and when it is high.

Crucially, there was no evidence of a foveal load effect in either experiment. Taken together with the findings of Zhang et al. [12] and Lv et al. [10], it appears that foveal load does not modulate either the spatial extent or the linguistic depth of parafoveal processing during Chinese reading. Despite this, in line with previous research [12,42], foveal load did modulate N + 1 skipping rates. Foveal load also modulated first-pass fixation times on N + 1 (a spillover effect, see Marx et al. [62]), adding further evidence that foveal load does not modulate the parafoveal preview, but does influence ongoing word processing and saccade targeting independently of the preview. The results of this study raise important theoretical considerations for models of eye movement control during reading [41,43,44]. The CRM [41] currently predicts foveal load effects during Chinese reading. In addition, in the EZ reader, spillover effects are hypothesized to arise from reduced parafoveal processing, an interpretation that is at odds with the current findings. In light of the growing evidence against such effects, this assumption may need to be revisited.

Additionally, comprehensive investigations of the spatial extent and linguistic depth of parafoveal processing remain absent in alphabetic languages, and so it will be important for future research to determine whether the lack of foveal load effects found in Chinese reflects a general lack of foveal load effects during reading or whether these findings are language specific. Lv et al. [10] speculate that the need to segment unspaced text may lead Chinese readers to develop more efficient processing through necessity, and so readers of Chinese may prioritize this parafoveal processing more than readers of alphabetic languages, as the consequences of not conducting parafoveal processing may be greater (e.g., segmentation failure). This prioritization may minimize the effect of foveal load relative to spaced languages. This possibility requires further investigation. An additional consideration concerns the structure of the target stimuli used in the current experiments. Here, both N + 1 and N + 2 were single-character words. This decision was made in order to maximize processing of N + 2 [15]. However, the majority of words in Chinese are composed of two characters (72%, Lexicon of common words in contemporary Chinese research team, 2008). Moreover, pre-processing of character N + 2 is more substantial when characters N + 1 and N + 2 form a single word (Xie et al., under review). Future research may seek to establish whether foveal load effects may be present for longer words and whether such effects depend on the lexical status of the characters, e.g., by comparing foveal load effects for character N + 3 when N + 3 is the final character of a three-character word and when character N + 3 is the first character of word N + 2 (following a two-character word N + 1). It is of course also important to acknowledge that despite conducting a well-powered study, our samples remain relatively small and homogeneous. We focused on skilled young adult readers, who may show particularly robust processing. To extrapolate to a more diverse range of readers, larger and more diverse sample sizes may be adopted in future studies to allow for a consideration of factors such as age and reading skill. Thus, how foveal load influences the type of information extracted during parafoveal processing remains a promising topic for future research.

## 5. Conclusions

In sum, the current study investigated whether foveal load influences the type of information extracted during parafoveal processing. We obtained a robust preview benefit of word N + 1 and N + 2; moreover, readers can acquire orthographic, phonological, and semantic information from N + 1. However, we found no evidence of a modulatory influence of foveal load on the linguistic depth of parafoveal processing, indicating that the foveal load effect is absent during natural Chinese reading.

## Figures and Tables

**Table 1:** Means and SDs for relatedness between the target and the four preview conditions in Experiment 1.

	Preview Conditions		
	Orthographic	Phonological	Semantic
Orthographic rating	5.6 (0.5)	1.3 (0.2)	1.3 (0.2)
Phonological rating	1.3 (0.2)	6.7 (0.3)	1.3 (0.2)
Semantic rating	1.3 (0.1)	1.3 (0.1)	6.0 (0.3)

**Table 2:** Means and SDs (in Parentheses) for the Eye Movement Measures for pretarget words (Word N) and target words (Word N + 1) in Experiment 1.

Measure	High Frequency					Low Frequency				
	ID	OR	PH	SE	PSE	ID	OR	PH	SE	PSE
Pretarget										
FFD	249 (51)	253 (53)	245 (49)	248 (67)	246 (48)	265 (57)	262 (61)	259 (55)	266 (62)	266 (70)
GD	271 (69)	286 (93)	280 (90)	277 (94)	286 (93)	315 (108)	316 (115)	309 (96)	319 (107)	310 (108)
TT	385 (135)	435 (165)	431 (210)	437 (182)	457 (191)	477 (192)	516 (201)	530 (271)	516 (232)	526 (250)
RPD	328 (118)	338 (128)	341 (136)	322 (129)	355 (128)	391 (162)	390 (160)	400 (198)	399 (207)	400 (159)
SKIP	0.29 (0.26)	0.30 (0.25)	0.31 (0.27)	0.30 (0.28)	0.29 (0.25)	0.25 (0.25)	0.26 (0.26)	0.24 (0.25)	0.27 (0.24)	0.28 (0.25)
Target										
FFD	260 (67)	332 (91)	338 (92)	332 (100)	352 (107)	271 (77)	334 (94)	336 (105)	351 (104)	339 (84)
GD	264 (69)	351 (101)	364 (106)	354 (106)	377 (111)	286 (89)	358 (106)	354 (121)	389 (140)	372 (106)
TT	310 (132)	404 (159)	404 (141)	416 (186)	424 (180)	347 (117)	421 (148)	429 (147)	467 (257)	447 (187)
RPD	330 (158)	425 (173)	472 (195)	456 (182)	460 (206)	401 (188)	458 (157)	455 (177)	495 (202)	486 (203)
SKIP	0.58 (0.24)	0.55 (0.28)	0.51 (0.25)	0.57 (0.25)	0.49 (0.27)	0.53 (0.24)	0.50 (0.26)	0.50 (0.26)	0.52 (0.24)	0.45 (0.24)

Note. ID = identical to the target word; OR = orthographically related to the target word, PH = phonologically related to target word, SE = semantically related to target word, PSE = pseudo-character unrelated to target word.

**Table 3:** Statistical Effects for the eye movement measures for word N and word N + 1 in Experiment 1.

Fixed Effect	Word n			Word n + 1		
	b	SE	t/z	b	SE	t/z
FFD						
Intercept	256.70	2.84	90.24	325.58	3.77	86.33
Frequency (High vs. Low)	15.78	2.67	5.91 *	4.00	4.51	0.89
Preview (PSE vs. ID)	2.09	3.72	0.56	82.67	6.76	12.24 *
Preview (PSE vs. OR)	2.50	3.72	0.67	11.41	7.11	1.60
Preview (PSE vs. PH)	5.85	3.72	1.57	7.28	6.56	1.11
Preview (PSE vs. SE)	3.13	3.92	0.80	2.21	6.65	0.33
Frequency × Preview (PSE vs. ID)	3.14	7.56	0.42	−17.74	12.60	−1.41
Frequency × Preview (PSE vs. OR)	6.05	7.44	0.81	−9.17	12.62	−0.73
Frequency × Preview (PSE vs. PH)	0.18	7.50	0.02	−12.36	12.75	−0.97
Frequency × Preview (PSE vs. SE)	1.25	8.69	0.14	−20.19	12.49	−1.62
GD						
Intercept	298.74	5.42	55.09	347.64	4.33	80.28
Frequency (High vs. Low)	35.83	5.25	6.83 *	11.31	5.16	2.19 *
Preview (PSE vs. ID)	11.95	6.86	1.74	102.51	7.78	13.18 *
Preview (PSE vs. OR)	−0.54	6.87	−0.08	19.62	8.18	2.40 *
Preview (PSE vs. PH)	6.22	7.19	0.87	12.08	7.45	1.62
Preview (PSE vs. SE)	5.92	7.15	0.83	2.64	7.36	0.36
Frequency × Preview (PSE vs. ID)	−16.40	13.73	−1.20	−16.32	14.46	−1.13
Frequency × Preview (PSE vs. OR)	−10.76	15.04	−0.72	−1.91	14.50	−0.13
Frequency × Preview (PSE vs. PH)	−9.91	13.93	−0.71	5.97	14.21	0.42
Frequency × Preview (PSE vs. SE)	−14.46	14.78	−0.98	−23.54	15.95	−1.48
TT						
Intercept	468.76	14.13	33.17	406.94	10.36	39.26
Frequency (High vs. Low)	83.99	12.15	6.91 *	29.24	6.36	4.60 *
Preview (PSE vs. ID)	64.59	12.50	5.17 *	115.59	11.75	9.83 *
Preview (PSE vs. OR)	12.83	11.21	1.14	21.59	9.79	2.21 *
Preview (PSE vs. PH)	12.08	13.85	0.87	21.43	10.16	2.11 *
Preview (PSE vs. SE)	14.78	11.61	1.27	−5.78	13.07	−0.44
Frequency × Preview (PSE vs. ID)	−10.12	22.55	−0.45	−13.72	20.55	−0.67
Frequency × Preview (PSE vs. OR)	−6.51	22.43	−0.29	15.70	19.86	0.79
Frequency × Preview (PSE vs. PH)	−16.15	27.93	−0.58	−5.66	19.44	−0.29
Frequency × Preview (PSE vs. SE)	−6.06	24.47	−0.25	−26.30	21.02	−1.25
RPD						
Intercept	366.31	8.62	42.51	444.40	11.58	38.38
Frequency (High vs. Low)	61.02	8.67	7.03 *	31.33	9.77	3.21 *
Preview (PSE vs. ID)	19.60	11.35	1.73	119.09	15.59	8.16 *
Preview (PSE vs. OR)	12.22	10.80	1.13	33.07	14.33	2.31 *
Preview (PSE vs. PH)	15.24	10.88	1.40	14.72	14.08	1.05
Preview (PSE vs. SE)	19.20	11.07	1.74	1.78	13.61	0.13
Frequency × Preview (PSE vs. ID)	−15.74	21.95	−0.72	−28.53	28.58	−1.00
Frequency × Preview (PSE vs. OR)	−15.01	21.90	−0.69	6.99	27.39	0.26
Frequency × Preview (PSE vs. PH)	−9.38	22.25	−0.42	45.07	27.92	1.62
Frequency × Preview (PSE vs. SE)	−35.47	23.13	−1.53	−14.29	27.19	−0.53
SKIP						
Intercept	−1.17	0.10	−11.50	0.10	0.06	1.50
Frequency (High vs. Low)	−0.18	0.00	−3.02 *	−0.19	0.05	−3.83 *
Preview (PSE vs. ID)	0.06	0.09	0.70	−0.40	0.09	−4.61 *
Preview (PSE vs. OR)	0.02	0.09	0.21	−0.27	0.08	−3.44 *
Preview (PSE vs. PH)	0.07	0.09	0.74	−0.19	0.08	−2.43 *
Preview (PSE vs. SE)	0.00	0.09	0.01	−0.34	0.08	−4.41 *
Frequency × Preview (PSE vs. ID)	0.17	0.18	0.96	0.00	0.15	0.01
Frequency × Preview (PSE vs. OR)	0.14	0.17	0.81	0.01	0.15	0.04
Frequency × Preview (PSE vs. PH)	0.34	0.18	1.93	−0.14	0.15	−0.91
Frequency × Preview (PSE vs. SE)	0.09	0.18	0.50	0.01	0.17	0.08

Note. Significant effects are indicated by ‘*’.

**Table 4:** Means and SDs for relatedness between the target and the four preview conditions in Experiment 2.

	Preview Conditions		
	Orthographic	Phonological	Semantic
Orthographic rating	5.3 (0.3)	1.3 (0.3)	1.5 (0.2)
Phonological rating	1.2 (0.1)	6.8 (0.1)	1.2 (0.1)
Semantic rating	1.1 (0.1)	1.0 (0.0)	5.5 (0.3)

**Table 5:** Means and SDs (in parentheses) for the eye movement measures for pretarget words (Word N) and target words (Word N + 2) in Experiment 2.

Measure	High Frequency					Low Frequency				
	ID	OR	PH	SE	PSE	ID	OR	PH	SE	PSE
Pretarget										
FFD	232 (47)	239 (57)	247 (65)	241 (50)	241 (60)	256 (60)	255 (54)	256 (53)	251 (58)	252 (61)
GD	252 (74)	260 (96)	271 (85)	262 (76)	259 (88)	292 (100)	291 (98)	284 (84)	285 (101)	284 (106)
TT	346 (119)	375 (173)	389 (153)	381 (140)	386 (163)	426 (165)	440 (175)	444 (166)	449 (196)	442 (173)
RPD	300 (103)	310 (127)	324 (137)	306 (114)	300 (117)	352 (132)	350 (140)	331 (99)	337 (152)	331 (129)
Skip	0.34 (0.27)	0.34 (0.26)	0.33 (0.25)	0.34 (0.28)	0.37 (0.26)	0.30 (0.26)	0.29 (0.24)	0.32 (0.27)	0.32 (0.26)	0.31 (0.26)
Target										
FFD	259 (73)	304 (96)	319 (90)	320 (102)	310 (90)	257 (62)	306 (94)	307 (85)	297 (89)	305 (89)
GD	263 (74)	316 (107)	330 (104)	329 (104)	323 (97)	261 (63)	321 (104)	319 (92)	306 (94)	318 (101)
TT	317 (111)	376 (154)	379 (128)	410 (198)	383 (137)	321 (119)	383 (149)	382 (128)	392 (145)	380 (127)
RPD	335 (200)	379 (150)	416 (158)	402 (170)	432 (172)	361 (174)	456 (214)	475 (218)	421 (169)	449 (177)
Skip	0.54 (0.25)	0.49 (0.24)	0.50 (0.26)	0.48 (0.26)	0.49 (0.25)	0.56 (0.25)	0.49 (0.25)	0.47 (0.25)	0.48 (0.26)	0.45 (0.25)

Note. ID = identical to the target word; OR = orthographically related to the target word, PH = phonologically related to target word, SE = semantically related to target word, PSE = pseudo-character unrelated to target word.

**Table 6:** Statistical Effects for the eye movement measures for word N and word N + 2 in Experiment 2.

Fixed Effect	Word n			Word n + 2		
	b	SE	t/z	b	SE	t/z
FFD						
Intercept	247.33	3.20	77.22	298.14	4.09	72.85
Frequency (High vs. Low)	17.54	3.53	4.97 *	−5.14	3.99	−1.29
Preview (PSE vs. ID)	1.99	3.73	0.54	48.70	6.10	7.99 *
Preview (PSE vs. OR)	0.06	3.90	0.01	3.37	6.47	0.52
Preview (PSE vs. PH)	−3.32	3.85	−0.86	−1.03	6.08	−0.17
Preview (PSE vs. SE)	−1.28	3.76	−0.34	−1.81	6.56	−0.28
Frequency × Preview (PSE vs. ID)	−12.05	7.47	−1.61	5.21	12.12	0.43
Frequency × Preview (PSE vs. OR)	−6.13	7.63	−0.80	−0.72	12.58	−0.06
Frequency × Preview (PSE vs. PH)	−3.61	7.91	−0.46	5.13	12.27	0.42
Frequency × Preview (PSE vs. SE)	−3.25	7.52	−0.43	11.67	13.29	0.88
GD						
Intercept	274.09	5.73	47.86	309.31	4.68	66.16
Frequency (High vs. Low)	31.16	5.88	5.30 *	−4.36	4.57	−0.96
Preview (PSE vs. ID)	−1.35	7.38	−0.18	58.47	6.72	8.70 *
Preview (PSE vs. OR)	−4.40	6.06	−0.73	2.01	7.20	0.28
Preview (PSE vs. PH)	−4.87	6.10	−0.80	−0.85	7.08	−0.12
Preview (PSE vs. SE)	−1.71	6.12	−0.28	1.67	7.36	0.23
Frequency × Preview (PSE vs. ID)	−11.78	12.22	−0.96	2.94	13.49	0.22
Frequency × Preview (PSE vs. OR)	−3.29	13.13	−0.25	−5.32	14.43	−0.37
Frequency × Preview (PSE vs. PH)	6.12	12.47	0.49	1.48	13.36	0.11
Frequency × Preview (PSE vs. SE)	2.66	12.24	0.22	9.20	14.20	0.65
TT						
Intercept	4.04	1.19	34.05	373.44	7.45	50.16
Frequency (High vs. Low)	6.54	1.07	6.12 *	−3.28	6.52	−0.50
Preview (PSE vs. ID)	2.91	1.04	2.80 *	64.45	10.15	6.35 *
Preview (PSE vs. OR)	8.33	1.10	0.76	2.16	12.21	0.18
Preview (PSE vs. PH)	−6.08	9.80	0.00	2.23	9.71	0.23
Preview (PSE vs. SE)	3.99	1.01	0.39	−19.31	12.06	−1.60
Frequency × Preview (PSE vs. ID)	−2.45	1.96	−1.25	−7.89	20.09	−0.39
Frequency × Preview (PSE vs. OR)	−1.48	1.96	−0.76	−7.25	20.84	−0.35
Frequency × Preview (PSE vs. PH)	−8.87	2.00	−0.44	−2.08	19.42	−0.11
Frequency × Preview (PSE vs. SE)	−1.12	2.08	−0.54	12.04	27.68	0.44
RPD						
Intercept	324.09	7.71	42.01	411.43	8.25	49.86
Frequency (High vs. Low)	31.84	7.24	4.40 *	41.94	9.46	4.44 *
Preview (PSE vs. ID)	−12.59	10.11	−1.25	94.82	13.61	6.97 *
Preview (PSE vs. OR)	−15.42	9.16	−1.68	17.78	13.45	1.32
Preview (PSE vs. PH)	−13.93	8.70	−1.60	−5.37	14.14	−0.38
Preview (PSE vs. SE)	−5.14	8.80	−0.58	25.62	13.76	1.86
Frequency × Preview (PSE vs. ID)	−22.03	18.81	−1.17	−9.66	26.37	−0.37
Frequency × Preview (PSE vs. OR)	−6.30	18.62	−0.34	−41.06	28.57	−1.44
Frequency × Preview (PSE vs. PH)	17.17	17.40	0.99	−28.97	28.21	−1.03
Frequency × Preview (PSE vs. SE)	0.86	17.53	0.05	3.55	25.56	0.14
SKIP						
Intercept	−0.90	0.10	−9.32	−0.01	0.06	−0.23
Frequency (High vs. Low)	−0.24	0.07	−3.63 *	−0.05	0.05	−0.93
Preview (PSE vs. ID)	0.14	0.09	1.61	−0.39	0.08	−4.96 *
Preview (PSE vs. OR)	0.16	0.09	1.76	−0.11	0.08	−1.33
Preview (PSE vs. PH)	0.08	0.09	0.88	−0.10	0.08	−1.35
Preview (PSE vs. SE)	0.08	0.08	0.92	−0.08	0.08	−0.96
Frequency × Preview (PSE vs. ID)	−0.06	0.18	−0.34	−0.30	0.16	−1.88
Frequency × Preview (PSE vs. OR)	0.05	0.17	0.28	−0.19	0.15	−1.27
Frequency × Preview (PSE vs. PH)	−0.22	0.17	−1.28	−0.04	0.16	−0.23
Frequency × Preview (PSE vs. SE)	−0.10	0.18	−0.58	−0.16	0.15	−1.04

Note. Significant effects are indicated by ‘*’.

## Data Availability

Experimental data and associated R code are available at https://osf.io/54rg2/. Accessed on 16 April 2024.

## References

[1] Rayner K. (1975). The Perceptual Span and Peripheral Cues in Reading. Cogn. Psychol..

[2] Rayner K. (1998). Eye Movements in Reading and Information Processing: 20 Years of Research. Psychol. Bull..

[3] Schotter E.R., Angele B., Rayner K. (2012). Parafoveal Processing in Reading. Atten. Percept. Psychophys..

[4] Henderson J.M., Ferreira F. (1990). Effects of Foveal Processing Difficulty on the Perceptual Span in Reading: Implications for Attention and Eye Movement Control. J. Exp. Psychol. Learn. Mem. Cogn..

[5] Veldre A., Andrews S. (2018). How Does Foveal Processing Difficulty Affect Parafoveal Processing during Reading?. J. Mem. Lang..

[6] Pan J., Yan M. (2022). Preview Frequency Effects in Reading: Evidence from Chinese. Psychol. Res..

[7] Bai X., Yan G., Liversedge S., Zang C., Rayner K. (2008). Reading Spaced and Unspaced Chinese Text: Evidence From Eye Movements. J. Exp. Psychol. Hum. Percept. Perform..

[8] Yan M., Sommer W. (2019). The Effects of Emotional Significance of Foveal Words on the Parafoveal Processing of N + 2 Words in Reading Chinese Sentences. Read. Writ..

[9] Yan M. (2015). Visually Complex Foveal Words Increase the Amount of Parafoveal Information Acquired. Vis. Res..

[10] Lv Y., Zhang L., Chen W., Xie F., Warrington K. (2023). The Influence of Foveal Load on Parafoveal Processing of N + 2 during Chinese Reading. Vis. Cogn..

[11] Vasilev M.R., Angele B. (2017). Parafoveal Preview Effects from Word N + 1 and Word N + 2 during Reading: A Critical Review and Bayesian Meta-Analysis. Psychon. Bull. Rev..

[12] Zhang M., Liversedge S., Bai X., Yan G., Zang C. (2019). The Influence of Foveal Lexical Processing Load on Parafoveal Preview and Saccadic Targeting during Chinese Reading Journal of Experimental Psychology: Human Perception and Performance. J. Exp. Psychol. Hum. Percept. Perform..

[13] Findelsberger E., Hutzler F., Hawelka S. (2019). Spill the Load: Mixed Evidence for a Foveal Load Effect, Reliable Evidence for a Spillover Effect in Eye-Movement Control during Reading. Atten. Percept. Psychophys..

[14] Vasilev M.R., Yates M., Prueitt E., Slattery T.J. (2021). Parafoveal Degradation during Reading Reduces Preview Costs Only When It Is Not Perceptually Distinct. Q. J. Exp. Psychol..

[15] He L., Ma W., Shen F., Wang Y., Wu J., Warrington K.L., Liversedge S.P., Paterson K.B. (2021). Adult Age Differences in Parafoveal Preview Effects during Reading: Evidence from Chinese. Psychol. Aging.

[16] Yan M., Richter E.M., Shu H., Kliegl R. (2009). Readers of Chinese Extract Semantic Information from Parafoveal Words. Psychon. Bull. Rev..

[17] Coltheart M., Rastle K., Perry C., Langdon R., Ziegler J. (2001). DRC: A Dual Route Cascaded Model of Visual Word Recognition and Reading Aloud. Psychol. Rev..

[18] Balota D.A., Pollatsek A., Rayner K. (1985). The Interaction of Contextual Constraints and Parafoveal Visual Information in Reading. Cogn. Psychol..

[19] Briihl D., Inhoff A.W. (1995). Integrating Information across Fixations during Reading: The Use of Orthographic Bodies and of Exterior Letters. J. Exp. Psychol. Learn. Mem. Cogn..

[20] Drieghe D., Rayner K., Pollatsek A. (2005). Eye Movements and Word Skipping During Reading Revisited. J. Exp. Psychol. Hum. Percept. Perform..

[21] White S.J. (2008). Eye Movement Control during Reading: Effects of Word Frequency and Orthographic Familiarity. J. Exp. Psychol. Hum. Percept. Perform..

[22] Miellet S., Sparrow L. (2004). Phonological Codes Are Assembled before Word Fixation: Evidence from Boundary Paradigm in Sentence Reading. Brain Lang..

[23] Tsai J.-L., Lee C.-Y., Tzeng O.J.L., Hung D.L., Yen N.-S. (2004). Use of Phonological Codes for Chinese Characters: Evidence from Processing of Parafoveal Preview When Reading Sentences. Brain Lang..

[24] Rayner K. (1986). Eye Movements and the Perceptual Span in Beginning and Skilled Readers. J. Exp. Child Psychol..

[25] Altarriba J., Kambe G., Pollatsek A., Rayner K. (2001). Semantic Codes Are Not Used in Integrating Information across Eye Fixations in Reading: Evidence from Fluent Spanish-English Bilinguals. Percept. Psychophys..

[26] White S.J., Bertram R., Hyönä J. (2008). Semantic Processing of Previews within Compound Words. J. Exp. Psychol. Learn. Mem. Cogn..

[27] Schotter E.R. (2013). Synonyms Provide Semantic Preview Benefit in English. J. Mem. Lang..

[28] Schotter E.R., Reichle E.D., Rayner K. (2014). Rethinking Parafoveal Processing in Reading: Serial-Attention Models Can Explain Semantic Preview Benefit and N+2 Preview Effects. Vis. Cogn..

[29] Schotter E.R., Lee M., Reiderman M., Rayner K. (2015). The Effect of Contextual Constraint on Parafoveal Processing in Reading. J. Mem. Lang..

[30] Schotter E.R., Jia A. (2016). Semantic and Plausibility Preview Benefit Effects in English: Evidence from Eye Movements. J. Exp. Psychol. Learn. Mem. Cogn..

[31] Schotter E.R. (2018). Reading Ahead by Hedging Our Bets on Seeing the Future. Psychology of Learning and Motivation.

[32] Andrews S., Veldre A. (2019). What Is the Most Plausible Account of the Role of Parafoveal Processing in Reading?. Lang. Linguist. Compass.

[33] Hohenstein S., Kliegl R. (2014). Semantic Preview Benefit during Reading. J. Exp. Psychol. Learn. Mem. Cogn..

[34] Rayner K., Schotter E.R. (2014). Semantic Preview Benefit in Reading English: The Effect of Initial Letter Capitalization. J. Exp. Psychol. Hum. Percept. Perform..

[35] Hoosain R. (1991). Psycholinguistic Implications for Linguistic Relativity: A Case Study of Chinese.

[36] Yan M., Luo Y., Inhoff A.W. (2014). Syllable Articulation Influences Foveal and Parafoveal Processing of Words during the Silent Reading of Chinese Sentences. J. Mem. Lang..

[37] Liu W., Inhoff A.W., Ye Y., Wu C. (2002). Use of Parafoveally Visible Characters during the Reading of Chinese Sentences. J. Exp. Psychol. Hum. Percept. Perform..

[38] Yan M., Risse S., Zhou X., Kliegl R. (2012). Preview Fixation Duration Modulates Identical and Semantic Preview Benefit in Chinese Reading. Read. Writ..

[39] Zhou W., Shu H., Miller K., Yan M. (2018). Reliance on Orthography and Phonology in Reading of Chinese: A Developmental Study: Orthography and Phonology in Chinese Reading. J. Res. Read..

[40] Wang A., Yeon J., Zhou W., Shu H., Yan M. (2016). Cross-Language Parafoveal Semantic Processing: Evidence from Korean–Chinese Bilinguals. Psychon. Bull. Rev..

[41] Li X., Pollatsek A. (2020). An Integrated Model of Word Processing and Eye-Movement Control during Chinese Reading. Psychol. Rev..

[42] Liu Y., Reichle E.D., Li X. (2016). The Effect of Word Frequency and Parafoveal Preview on Saccade Length during the Reading of Chinese. J. Exp. Psychol. Hum. Percept. Perform..

[43] Reichle E.D., Liversedge S.P., Gilchrist I., Everling S. (2011). Serial-Attention Models of Reading. The Oxford Handbook of Eye Movements.

[44] Engbert R., Kliegl R., Liversedge S.P., Gilchrist I., Everling S. (2011). Parallel Graded Attention Models of Reading. The Oxford Handbook of Eye Movements.

[45] Taylor H.R. (1978). Applying New Design Principles to the Construction of an Illiterate E Chart. Am. J. Optom. Physiol. Opt..

[46] Westfall J., Kenny D.A., Judd C.M. (2014). Statistical Power and Optimal Design in Experiments in Which Samples of Participants Respond to Samples of Stimuli. J. Exp. Psychol. Gen..

[47] Cohen J. (1962). The Statistical Power of Abnormal-Social Psychological Research: A Review. J. Abnorm. Soc. Psychol..

[48] Cai Q., Brysbaert M. (2010). SUBTLEX-CH: Chinese Word and Character Frequencies Based on Film Subtitles. PLoS ONE.

[49] Slattery T.J., Angele B., Rayner K. (2011). Eye Movements and Display Change Detection during Reading. J. Exp. Psychol. Hum. Percept. Perform..

[50] Drieghe D., Chan Seem R. (2022). Parafoveal Processing of Repeated Words during Reading. Psychon. Bull. Rev..

[51] Baayen R.H., Davidson D.J., Bates D.M. (2008). Mixed-Effects Modeling with Crossed Random Effects for Subjects and Items. J. Mem. Lang..

[52] Bates D., Mächler M., Bolker B., Walker S. (2015). Fitting Linear Mixed-Effects Models Using Lme4. J. Stat. Softw..

[53] R Core Team (2017). R: A Language and Environment for Statistical Computing.

[54] Barr D.J., Levy R., Scheepers C., Tily H.J. (2013). Random Effects Structure for Confirmatory Hypothesis Testing: Keep It Maximal. J. Mem. Lang..

[55] Venables W.N., Ripley B.D., Chambers J., Eddy W., Härdle W., Sheather S., Tierney L. (2002). Modern Applied Statistics with S.

[56] Yan G., Tian H., Bai X., Rayner K. (2006). The Effect of Word and Character Frequency on the Eye Movements of Chinese Readers. Br. J. Psychol..

[57] Li X., Bicknell K., Liu P., Wei W., Rayner K. (2014). Reading Is Fundamentally Similar across Disparate Writing Systems: A Systematic Characterization of How Words and Characters Influence Eye Movements in Chinese Reading. J. Exp. Psychol. Gen..

[58] Pan J., Yan M., Laubrock J. (2020). Semantic Preview Benefit and Cost: Evidence from Parafoveal Fast-Priming Paradigm. Cognition.

[59] Yin B. (1994). Xian Dai Han Zi.

[60] Yan M., Kliegl R., Shu H., Pan J., Zhou X. (2010). Parafoveal Load of Word N+1 Modulates Preprocessing Effectiveness of Word N+2 in Chinese Reading. J. Exp. Psychol. Hum. Percept. Perform..

[61] Yu L., Cutter M.G., Yan G., Bai X., Fu Y., Drieghe D., Liversedge S.P. (2016). Word *n* + 2 Preview Effects in Three-Character Chinese Idioms and Phrases. Lang. Cogn. Neurosci..

[62] Marx C., Hawelka S., Schuster S., Hutzler F. (2017). Foveal Processing Difficulty Does Not Affect Parafoveal Preprocessing in Young Readers. Sci. Rep..

